# Age-Related P-Glycoprotein Expression in the Intestine and Affecting the Pharmacokinetics of Orally Administered Enrofloxacin in Broilers

**DOI:** 10.1371/journal.pone.0074150

**Published:** 2013-09-16

**Authors:** Mengjie Guo, Shamsuddin Bughio, Yong Sun, Yu Zhang, Lingling Dong, Xiaohua Dai, Liping Wang

**Affiliations:** Laboratory of Veterinary Pharmacology and Toxicology, College of Veterinary Medicine, Nanjing Agricultural University, Nanjing, Jiangsu Province, P. R. China; University of Strathclyde, United Kingdom

## Abstract

Bioavailability is the most important factor for the efficacy of any drug and it is determined by P- glycoprotein (P-gp) expression. Confirmation of P-gp expression during ontogeny is needed for understanding the differences in therapeutic efficacy of any drug in juvenile and adult animals. In this study, Abcb1 mRNA levels in the liver and intestine of broilers during ontogeny were analysed by RT qPCR. Cellular distribution of P-gp was detected by immunohistochemstry. Age-related differences of enrofloxacin pharmacokinetics were also studied. It was found that broilers aged 4 week-old expressed significantly (*P*<0.01) higher levels of P-gp mRNA in the liver, jejunum and ileum, than at other ages. However, there was no significant (*P*>0.05) age-related difference in the duodenum. Furthermore, the highest and lowest levels of Abcb1 mRNA expression were observed in the jejunum, and duodenum, respectively. P-gp immunoreactivity was detected on the apical surface of the enterocytes and in the bile canalicular membranes of the hepatocytes. Pharmacokinetic analysis revealed that the 8 week-old broilers, when orally administrated enrofloxacin, exhibited significantly higher C_max_ (1.97 vs. 0.98 μg•ml^-1^, *P*=0.009), AUC(14.54 vs. 9.35 μg•ml^-1^•h, *P*=0.005) and Ka (1.38 vs. 0.43 h^-1^, *P*=0.032), as well as lower T_peak_ (1.78 vs. 3.28 h, *P*=0.048) and T_1/2ka_ (0.6 vs. 1.64 h, *P*=0.012) than the 4 week-old broilers. The bioavailability of enrofloxacin in 8 week-old broilers was increased by 15.9%, compared with that in 4 week-old birds. Interestingly, combining verapamil, a P-gp modulator, significantly improved pharmacokinetic behaviour of enrofloxacin in all birds. The results indicate juvenile broilers had a higher expression of P-gp in the intestine, affecting the pharmacokinetics and reducing the bioavailability of oral enrofloxacin in broilers. On the basis of our results, it is recommended that alternative dose regimes are necessary for different ages of broilers for effective therapy.

## Introduction

ABC transporters can carry a wide range of substrates across biological membranes, by hydrolysing ATP as an energy source [1]. Although these transporters are prone to over-expression in tumours, their expression is widespread throughout many normal tissues such as the liver, kidney and intestine in mammals [2,3,4,5]. P-glycoprotein (P-gp, 170 kDa), a multi-drug resistance gene (also known as *Abcb1* gene) product, is the most important transporter for clinically relevant drugs and is the focus of this study. The data from knockout mice suggest that intestinal P-gp may play a direct clinical role in the disposition of orally administered drugs and in explaining certain clinical drug-drug interactions [6,7,8]. The expression of efflux transporters is regulated in a highly dynamic manner [9]. Regarding the physiological changes in P-gp expression, it has been recently suggested that age might affect basal P-glycoprotein levels in human and mice [10,11,12]. Age-related differences have been observed in the pharmacokinetics of the P-gp substrates digoxin [13] and fexofenadine [14]. In poultry, only a few studies [15,16] have partially characterised the expression of P-gp in tissues of chickens and turkeys. There is increasing recognition for the role of P-gp in veterinary therapy [17]. However, it is unknown whether age affects P-gp expression in broilers, because P-gp expression affects the pharmacokinetics of many orally administered drugs, including anti-parasitic and chemotherapeutic drugs in ruminant species [18]. In addition, P-gp modulators can profoundly affect the plasma disposition of chemotherapeutic drugs. Fluoroquinolones have been successfully used for the treatment of colibacillosis and mycoplasma infection in poultry [19,20]. Some of them, such as danofloxacin and gatifloxacin, have proven to be the substrates for ABC transporters [21,22,23,24]. Enrofloxacin is also one of the most extensively used therapeutic agents in poultry. However, very little is known about the role of ABC transporters in the pharmacokinetics of orally administered enrofloxacin in chickens, especially in broilers at different ages.

This study investigated P-gp expression in the liver and small intestines in broilers at different ages (from young adults to slaughter stage), the effect of different P-gp expression levels on enrofloxacin pharmacokinetics in broilers aged 4 and 8 weeks, and also using the P-gp modulator verapamil. This study reveals that juvenile broilers have higher expression of P-gp in the intestine, which affected pharmacokinetics by reducing the bioavailability of orally administered enrofloxacin in the broilers.

## Materials and Methods

### Reagents and drugs

Mouse monoclonal anti-P-gp (C219) and rabbit polyclonal anti-P-gp antibodies, used for immunohistochemistry, were from Covance (Princeton, New Jersey, USA) and Biossn (Wuhan, Hubei, China), respectively. Rabbit anti-rat IgG-horseradish peroxidase (HRP) was purchased from Boster (Wuhan, Hubei, China). Enrofloxacin hydrochloride (ENRO, bulk drug) was a gift from the China Institute of Veterinary Drug Control (Beijing, China). Verapamil was purchased from Sigma (St. Louis, MO, USA). All other compounds used were reagent grade from local vendors.

### Animals and sample collection

Total 20 one-day-old Ross broilers were purchased from a commercial hatchery (Wuxi, China) and raised under standard conditions of light and temperature. Feed (without antibiotics and coccidiostats) and water were provided *ad libitum*. Tissue samples (duodenum, jejunum, ileum and liver) were collected at 2, 4, 6 and 8 weeks of age, respectively. Five randomly chosen birds were sacrificed by decapitation at predetermined time intervals. After being sub-packaged in aliquots and snap-frozen in liquid nitrogen, all samples were stored at -70°C until real-time RT-PCR analysis was performed. The use of the birds followed the protocol approved by Nanjing Agricultural University Animal Care and Use Committee.

### RNA isolation and mRNA quantification

Real time RT-PCR was used to detect the mRNA expression level in liver and different parts of intestine in broilers at different ages. Total RNA was isolated from individual tissues of all birds using Trizol Reagent (Takara, Tokyo, Japan) according to the manufacturer’s instructions. Total RNAs were treated with 100 U DNase I (RNase Free, Takara, Tokyo, Japan) for 30 min at 37°C to ensure that all total RNA was free of genomic DNA contamination. The total RNA concentration was then quantified by Nanodrop photometer (ND-1000 Spectrophotometer, Rockland, DE, USA). Ratios of absorption (260/280 nm) of all preparations were between 1.8 and 2.0. Each RNA sample was subjected to electrophoresis on a 1.4% agarose formaldehyde gel to verify its integrity. Single-stranded cDNAs were synthesised and real-time PCR was performed, as previously described [25]. Negative controls involved the omission of RNA from the RT reactions and amplification with specific primer/probe sets to confirm the lack of genomic DNA contamination. Primers specific for P-gp and β-actin were designed as described [26] and commercially synthesised for real-time PCR analysis. Chicken β-actin was chosen as a housekeeping gene for normalisation, based on experiments showing stable expression of β-actin mRNA in the small intestine and liver in the broilers. The PCR products were sequenced to validate the identity of the amplicons. The 2 ^− ΔΔCt^ method [27] was used to analyse the real-time RT-PCR data.

### Immunohistochemistry

To localise P-gp protein expression in liver and small intestine in broilers, immunohistochemistry was performed. As the highest expression of P-gp mRNA was detected in broilers at 4 weeks of age by real-time RT-PCR analysis, the liver and small intestinal samples were randomly collected from 5 broilers aged 4 weeks. Immunostaining was performed on 5 µm paraffin tissue sections mounted on APES-coated slides. After deparafﬁnisation in xylene and rehydration through graded ethanol, endogenous peroxidase activity was blocked with 3% (v/v) H_2_O_2_ for 15 min. Then, antigen retrieval was performed by heating the sections in 0.01 M citrate buffer (pH 6.0), and nonspecific protein binding sites were blocked with 5% bovine serum albumin (BSA) at 37°C for 30 min. Thereafter, the tissue-sections were incubated overnight at 4°C with the primary antibody (monoclonal anti-P-gp, diluted 1:20; or rabbit anti-P-gp, diluted 1:200). After washing with PBS, the secondary antibody was applied and samples were incubated for 45 min at 37°C. A streptavidin-biotin-complex was added and the specimens were incubated for a further 30 min at 37°C. The P-gp immunoreactivity was visualised with DAB staining, according to the supplier’s instructions. Finally, sections were counterstained with haematoxylin, dehydrated and cleared with xylene and coated with neutral balsam. Sections treated as above, but without the primary antibody, served as negative controls. Monoclonal anti-P-gp (C219) was used to detect P-gp in liver, and intestine. Two semi-quantitative measurements for P-glycoprotein staining were performed by two experimental pathologists in a double-blind analysis by a light microscope (BX45-DP72; OLYMPUS, Tokyo, Japan) equipped with Plan Apo objectives connected to a CCD camera (U-TV0.63XC; OLYMPUS, Tokyo, Japan). The area and IOD labelled for P-glycoprotein (labelled surface area) was analysed in the liver, kidney and small intestinal using a computer-assisted image analysis system (Image-Pro Plus 4.1 software).

### Experiments with enrofloxacin in broilers

In total, forty 4 week-old and forty 8 week-old healthy broilers were randomly divided into four groups. The first group received a single dose of 10 mg/kg body weight (b. w.) of enrofloxacin orally through crop tube gavage. The second group was first orally administrated with verapamil (15 mg/kg b. w.) and then enrofloxacin (10 mg/kg b. w.). The third group received a single dose of 10 mg/kg b.w. of enrofloxacin intravenously (i.v.) through the left brachialis vein. The fourth group was first orally administrated with verapamil (15 mg/kg b. w.) and then injected with enrofloxacin (10 mg/kg b. w.). Blood samples were collected from the right brachialis vein prior to treatment and at 5, 15, 45 min and 1, 2, 4, 6, 8, 12 h in plastic tubes after the administration of enrofloxacin in each group. The blood samples were immediately centrifuged at 3 000 g for 15 min and stored at -20°C until analysis.

### Assay for enrofloxacin in serum by HPLC

The serum concentrations of enrofloxacin were detected on an Agilent 1200 high-performance liquid chromatography (HPLC) system as described previously with minor modifications [28,29]. Briefly, the samples were thawed at room temperature and centrifuged at 2000 g for 5 min, the supernatant (0.5 ml) was applied to acetonitrile and the organic and water phases were separated by centrifugation. The organic phase was evaporated to dryness under a nitrogen stream and the residue was resuspended with mobile phase solution. Twenty microliters of the mixture were injected into the HPLC column. The composition of the mobile phase was 0.1M phosphoric acid (adjust pH to 3.0 with triethylamine)/acetonitrile (84:16). Chromatographic separations were performed on Kromasil C_18_ HPLC Columns (5 μm, 25 cm×4.6 mm). The flow rate of the mobile phase was set to 0.85 mL/min. UV absorbance was measured at 278 nm. Drugs were quantified by measuring the peak area.

**Figure 1:**
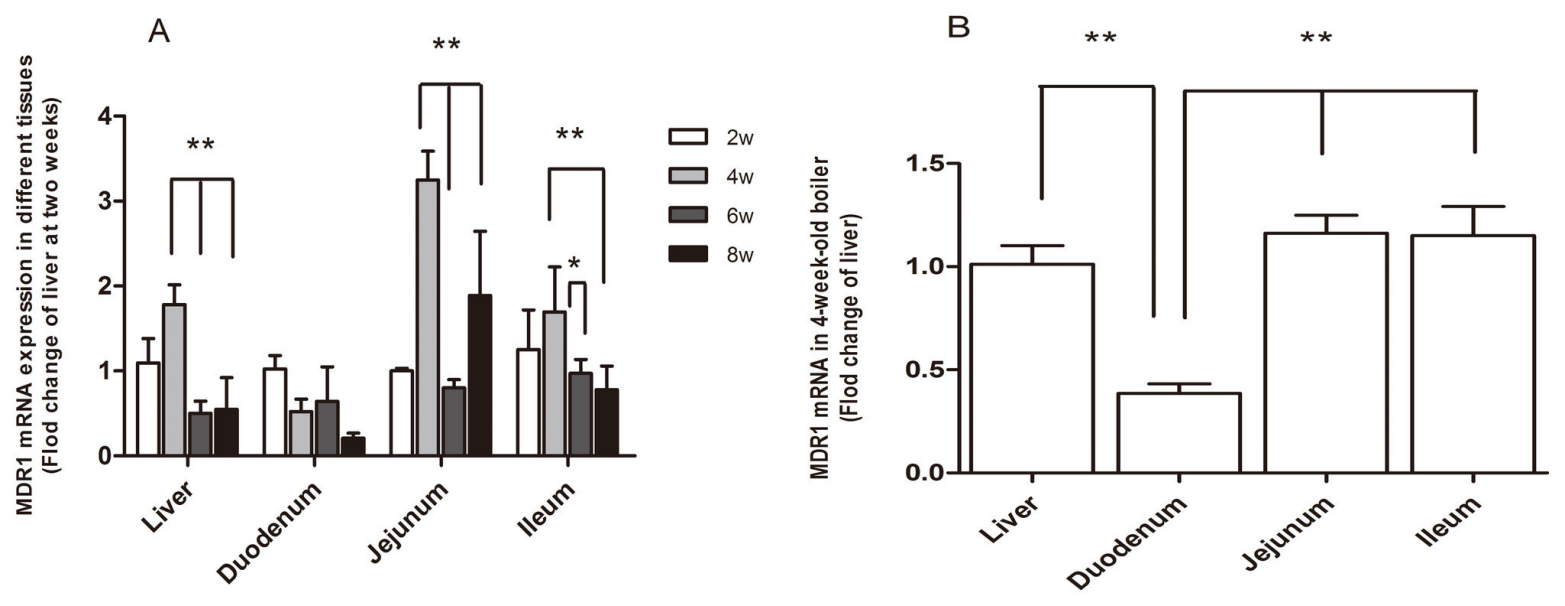
Expression of P-gp mRNA in broilers at different ages. (A) Expression levels of P-gp mRNA in liver, jejunum, ileum and duodenum in broilers at indicated ages, as detected by real time RT PCR. (B) Relative comparison of expression levels of P-gp mRNA in jejunum, ileum and duodenum in 4 week-old broilers, as detected by real time RT PCR. β-actin was used as a reference gene for normalization (n=5). ** difference between ages of tissues (*P*<0.01).

**Figure 2:**
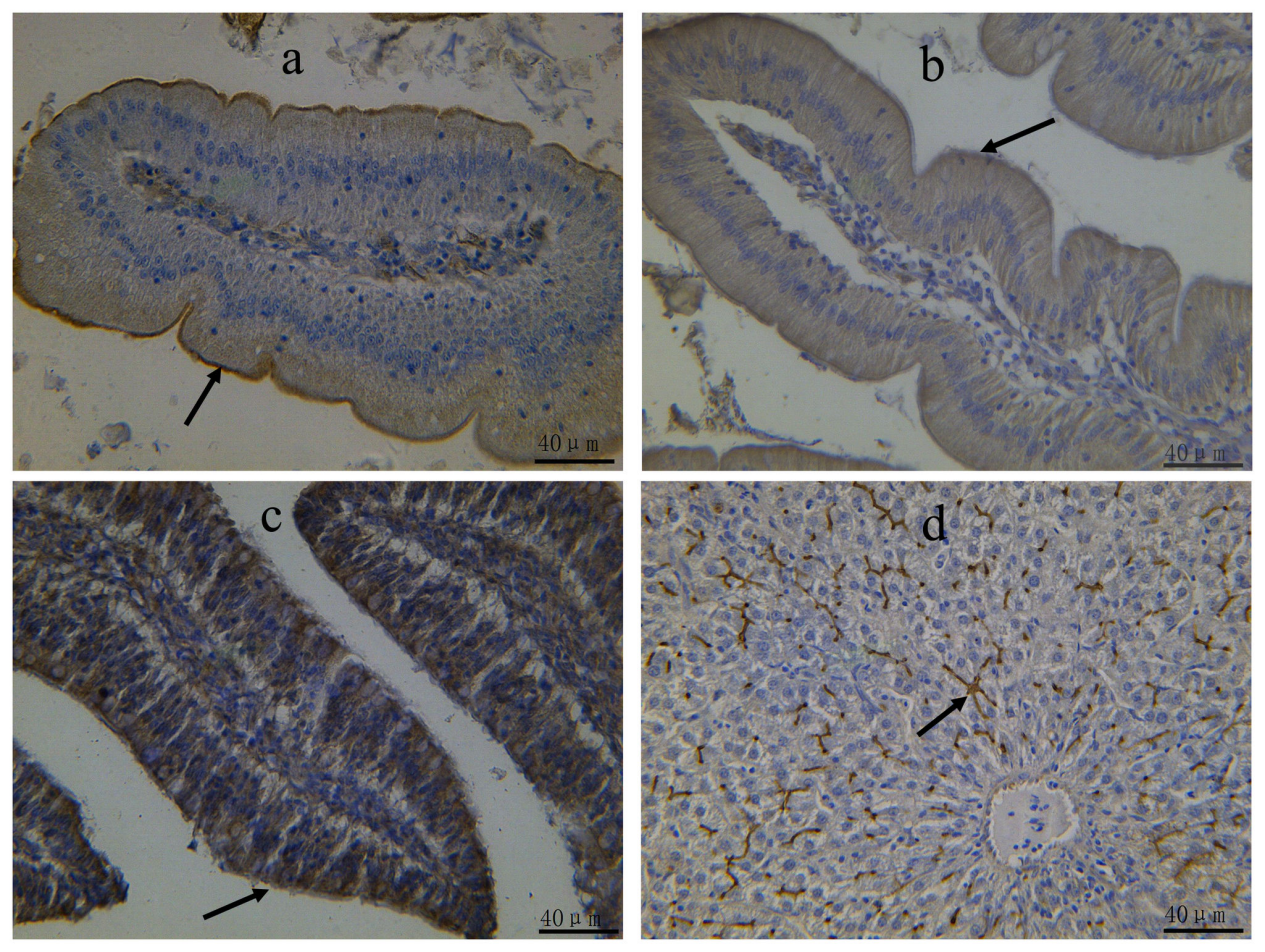
Immunohistochemical staining of P-gp in the liver and intestines of 4 week-old broilers. Rabbit anti-P-gp polyclonal antibodies and mouse monoclonal anti-P-gp antibody (C219) were used to detect P-gp in duodenum (a), jejunum (b), ileum (c) and liver (d) of broilers, respectively. Bar = 40 μm.

### Pharmacokinetic analysis

Pharmacokinetic calculations were performed on each individual set of data using 3p97 practical pharmacokinetic software (Version 97, Chinese Pharmacologic Association, Beijing, China). The best fit was determined according to the Akaike’s Information Criterion. The area under the concentration-time curve (AUC) was calculated according to the linear trapezoidal method.

### Data analysis

All data were presented as mean ± standard error (S.E.), and analysed by one-way ANOVA using SPSS 16.0 for Windows followed by a least-significant difference (LSD) test for individual comparisons. Values of mRNA abundance were expressed as the fold-change relative to the average value of one group. Pharmacokinetic parameters of enrofloxacin were analysed using *t*-test for independent-samples. The significance level was set at *P* < 0.05.

## Results

### Age-dependent mRNA expression in liver and small intestines in broilers

With regard to P-gp expression in broilers during ontogeny, first, we detected the mRNA expression of Abcb1 in broilers at ages from Day 1 to Day 7. However, to our surprise, the expression level of P-gp mRNA was very low in the liver or small intestines from those very young birds (data not shown). Therefore, we focused on studies of broilers aged from 2 to 8 weeks. According to the real-time RT-PCR results, the patterns of mRNA expression in the liver and small intestines (except duodenum) were age-dependent. As shown in Figure 1A, the broilers at 4 weeks of age expressed significantly higher P-gp mRNA levels in the liver, jejunum and ileum, than at other ages (*P*<0.01). However, no significant age-related difference was detected in P-gp mRNA expression in the duodenum (*P*>0.05), although variation was observed within each age group. After further analysing the intestinal samples of 4 week-old broilers, the highest expression level of Abcb1 mRNA was observed in the jejunum, while the lowest expression level of Abcb1 mRNA was seen in the duodenum (Figure 1B).

**Figure 3:**
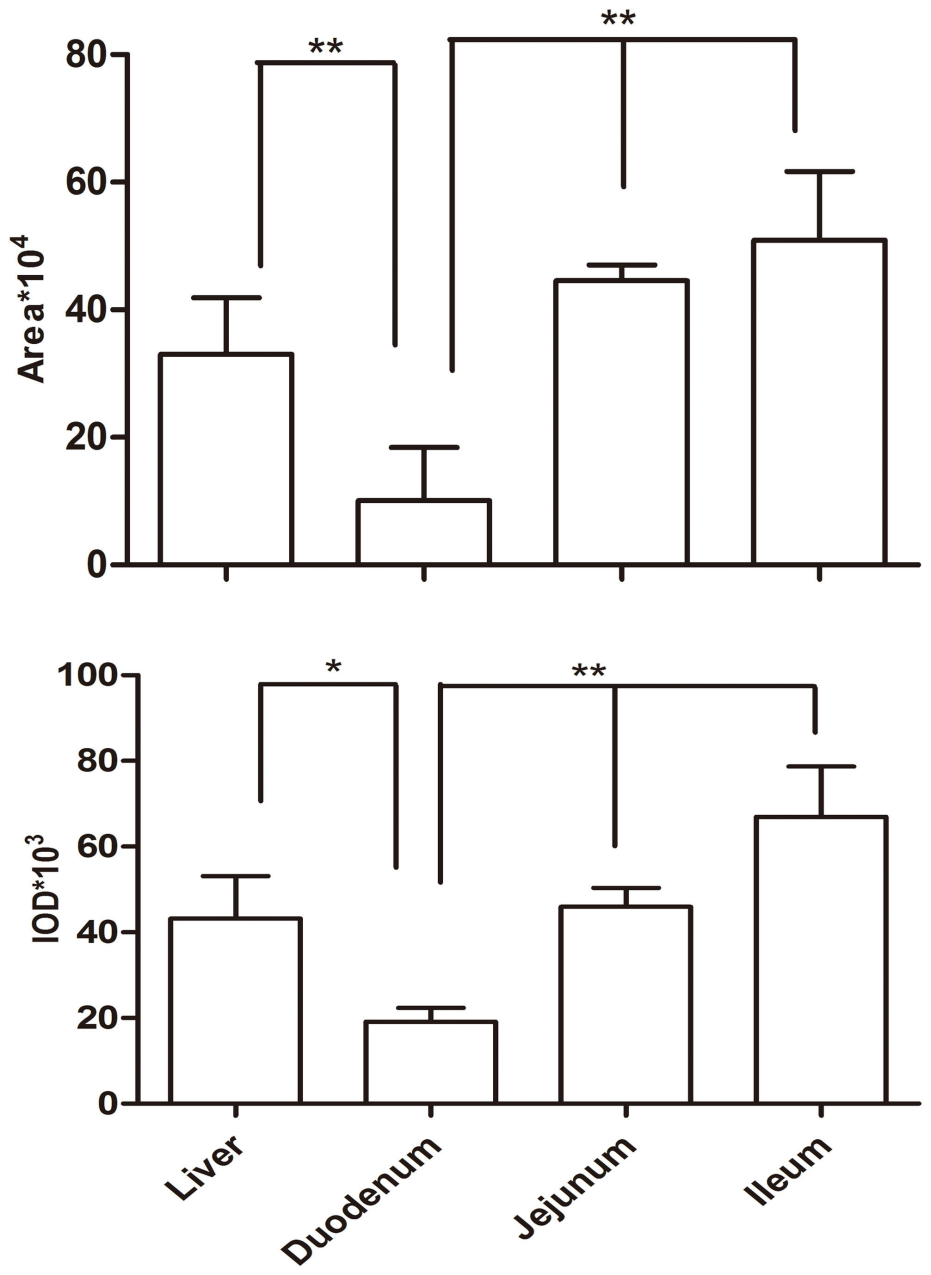
Semi-quantification of P-gp in liver and intestines of 4 week-old broilers. The intensity of specific P-gp staining was evaluated by measuring the IOD, area of positive staining using the Image-Pro Plus 4.1. software. Data were shown as mean ± S.E. (n=5) *P<0.05, **P<0.01.

### The cellular localisation and quantification of P-gp in liver and small intestines of broilers

The expression of P-gp protein in the liver and intestine of broilers (4 weeks of age) was further investigated by immunohistochemical studies using the P-gp antibody. No background staining was observed in the negative controls (data not shown). As shown in Figure 2, strong staining was detected in the liver, duodenum, jejunum or ileum. In the duodenum and jejunum, P-gp immunoreactivity was observed on the apical surface of the enterocytes. However, in the ileum, intense staining was visualised in the cytoplasm of the enterocytes. In liver, marked P-gp immunostaining was observed in the bile canalicular membranes of the hepatocytes. To validate our immunohistochemistry results, we semi-quantified the stained liver and small intestine using Image-Pro Plus 4.1 software. After further analysing the intestinal samples of 4 week-old broilers, the highest expression level of P-gp was observed in the jejunum (Figure 3), whereas the lowest expression level of P-gp was seen in the duodenum. Quantification of P-gp staining provided additional validity to our studies from the protein level, which is coincident with the trend of mRNA expression level in the tissues of 4 week-old broilers.

### Pharmacokinetics of enrofloxacin with a single application in broilers at different ages

**Figure 4:**
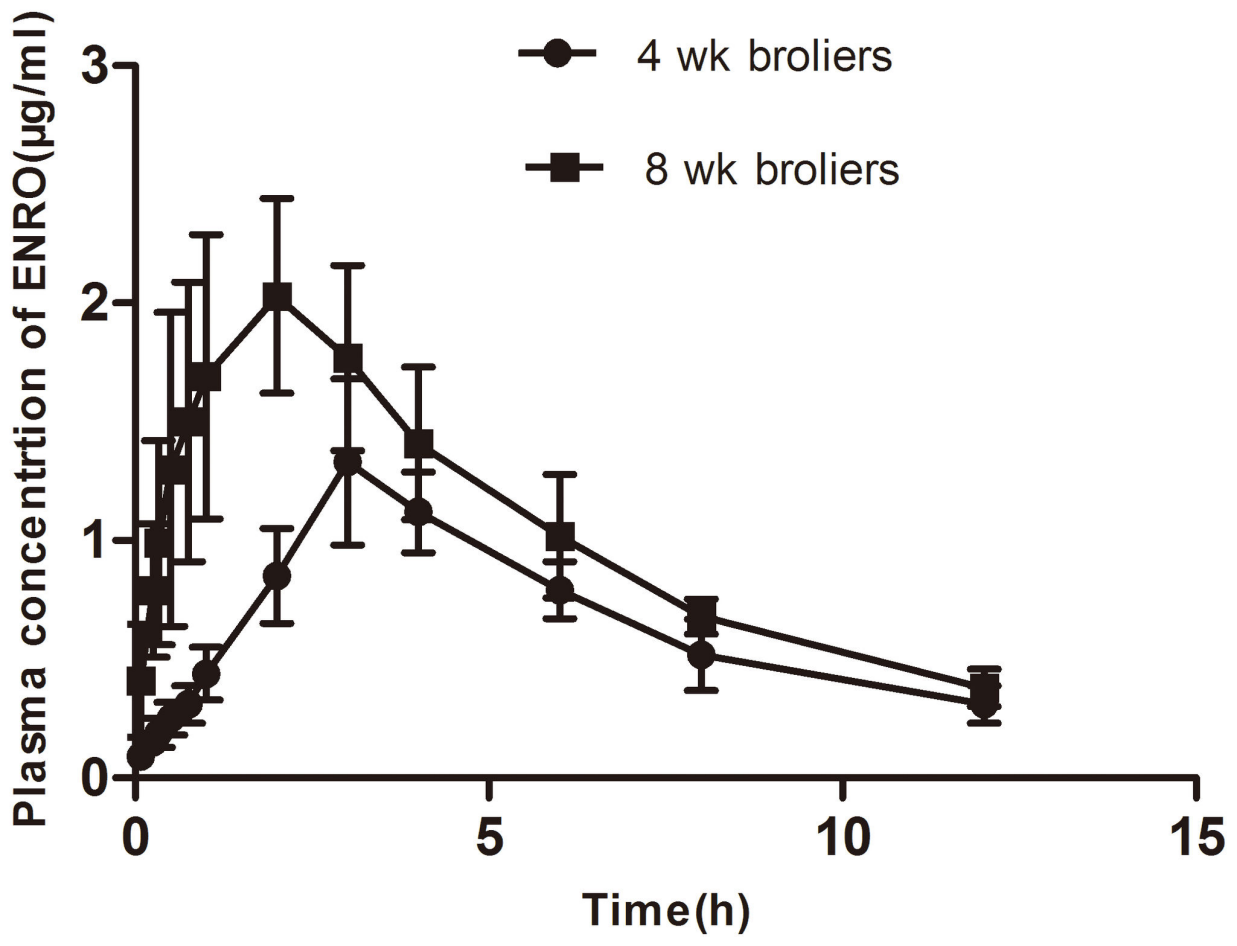
Plasma concentration-time profiles of orally administered enrofloxacin (10 mg/kg b.w.) in the broilers of 4 and 8 week old. Data represent mean ± S.E. (n=10).

To determine whether P-gp expression level affects the pharmacokinectics of enrofloxacin, the broilers at 4 and 8 weeks of age were selected, based on the highest and lowest levels of P-gp mRNA expression in the liver and intestines of the birds. The plasma concentration-time profiles of enrofloxacin after a single oral administration of enrofloxacin (10 mg/kg b.w.) in broilers at different ages are illustrated in Figure 3, and the relevant pharmacokinetic parameters are shown in Table 1. The results showed that there was a significant difference between the two groups (4 and 8 week-old broilers). As shown in Figure 4, enrofloxacin was detectable in plasma at the first sampling point (0.083 h) in all broilers. The 8 week-old broilers, when orally administered enrofloxacin, exhibited a significantly higher C_max_ (1.97 vs. 0.98 μg•mL^-1^, *P*=0.009), AUC (14.54 vs. 9.35 μg•mL^-1^•h, *P*=0.005) and K_a_ (1.38 vs. 0.43 h^-1^, *P*=0.032), as well as a lower T_peak_ (1.78 vs. 3.28 h, *P*=0.048) and T_1/2ka_ (0.6 vs. 1.64 h, *P*=0.012), compared with the 4 week-old broilers. In addition, the bioavailability of enrofloxacin in 8 week-old broilers was increased by 15.9% compared with that in 4 week-old birds. However, when enrofloxacin was i.v. administered, there were no significant differences of the main parameters between the two groups of broilers (Table 1). These results suggest that the higher expression of P-gp will possibly affect the pharmacokinetics of enrofloxacin, leading to decreased bioavailability of enrofloxacin in the 4 week-old broilers.

**Table 1:** Parameters of enrofloxacin, both orally and i.v. administered (10 mg/kg), in 4 and 8 week-old broilers (mean ± S.E., n=10).

Parameters	4 week old broilers	8 week old broilers
*Oral administration*		
*K*a (h^-1^)	0.43±0.09	1.38±0.7^*^
*t*_1/2ka_ (h)	1.64±0.33	0.6±0.27^**^
T_peak_ (h)	3.28±0.35	1.78±0.4^*^
C_max_ (μg•mL^-1^)	0.98±0.1	1.97±0.54^**^
AUC (μg•mL^-1^•h)	9.35±1.12	14.54±2.3^**^
*t*_1/2ke_ (h)	3.36±0.40	3.79±1.24
*Cl/f* (mL/min)	1.08±0.12	0.7±0.1
*i.v. administration*		
*t*_1/2_ (h)	4.52±0.47	5.23±1.5
AUC (μg•mL^-1^•h)	28.1±1.00	29.55±1.23
*Cll/f* (mL/min)	0.34±0.05	0.33±0.013
F (%)	33.3	49.2

* *p*< 0.05, ** *p* < 0.01 significant difference vs. 4 week old broilers. *K*_*a*_: absorption constant; *t*_1/2ka_: absorption half-life; T_peak_: time to reach peak concentration; C_max_: peak concentration; AUC: area under the plasma concentration-time curve from zero to time infinity; F: bioavailability

**Figure 5:**
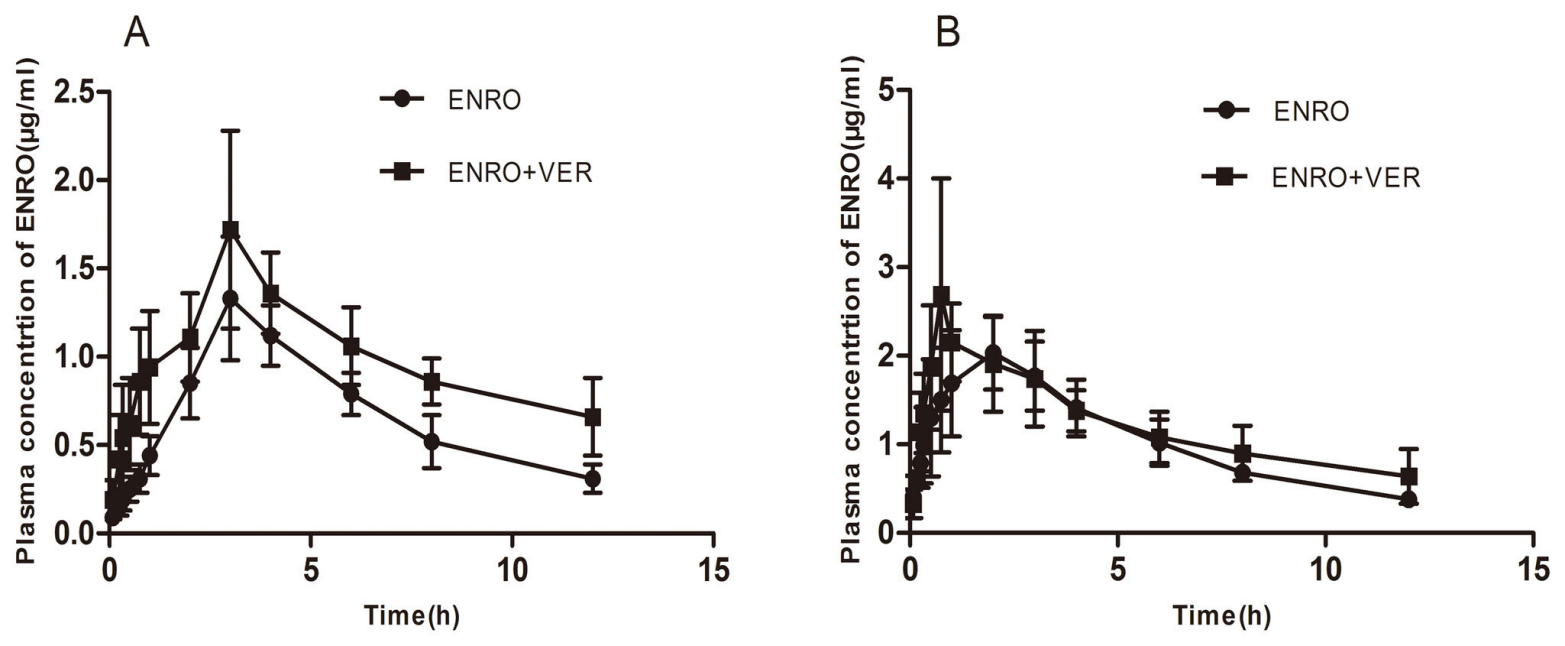
Plasma concentration-time profiles of orally administered enrofloxacin (10 mg/kg b.w.) in the presence and absence of verapamil (15 mg/kg b.w.) in the broilers of 4 week old (A) and 8 week old (B). Data represent mean ± S.E. (n=10).

**Figure 6:**
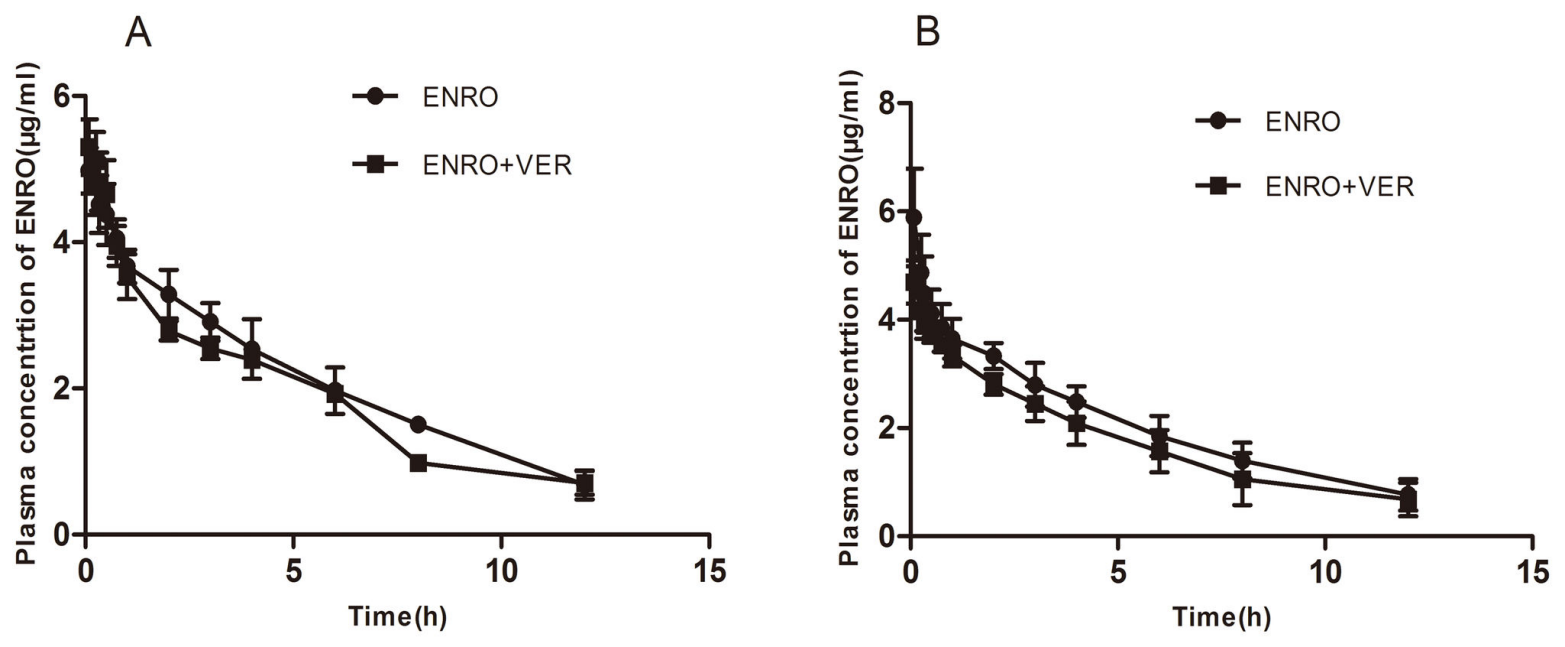
Plasma concentration-time profiles of i.v. treated enrofloxacin (10 mg/kg b.w.) in the presence of verapamil (15 mg/kg b.w.) in the broilers aged 4 weeks (A) and 8 weeks old (B). Data represent mean ± S.E. (n=10).

### Effect of verapamil on pharmacokinetics of orally administrated enrofloxacin

To substantiate the effect of P-gp expression level on the pharmacokinetics of enrofloxacin in broilers, verapamil, an inhibitor of P-gp expression [30], was employed. The mean plasma concentration-time profiles of oral enrofloxacin (10 mg/kg b. w.) in the presence or absence of oral verapamil (15 mg/kg b.w.) are shown in Figure 5. The relevant pharmacokinetic parameters are also listed in Table 2. The combination of enrofloxacin and verapamil caused significant changes in the pharmacokinetic behaviour of enrofloxacin. Compared to enrofloxacin alone, the combination of enrofloxacin/verapamil significantly increased the AUC _0-∞_ of enrofloxacin by 2.16- and 1.69-fold (*P*<0.01), and the C_max_ of enrofloxacin by 1.46- and 1.17-fold (*P*<0.05) in 4 and 8 week-old broilers, respectively. Also, the T_1/2ka_ and T_peak_ of enrofloxacin were lower after the co-administration of verapamil (*P*<0.05) in both 4 and 8 week-old broilers. The results support the notion that the level of P-gp profoundly impacts the pharmacokinetics of orally administered enrofloxacin in broilers.

**Table 2:** Parameters of enrofloxacin, both orally and i.v. administered (10 mg/kg), in 4 and 8 week-old broilers in the presence and absence of verapamil (15 mg/kg) (mean ± S.E., n=10).

Parameters	4 week old broilers		8 week old broilers	
	ENRO	ENRO+VER	ENRO	ENRO+VER
*Oral administration*				
*K*a (h^-1^)	0.43±0.09	1.02±0.53	1.38±0.7	2.98±0.57^#^
*t*_1/2ka_ (h)	1.64±0.33	0.79±0.31^*^	0.6±0.27	0.24±0.04^#^
T_peak_ (h)	3.28±0.35	2.86±0.93	1.78±0.4	1.11±0.29^#^
C_max_ (μg•mL^-1^)	0.98±0.1	1.43±0.15^*^	1.97±0.54	2.3±0.74^#^
AUC (μg•mL^-1^•h)	9.35±1.12	20.24±4.49^**^	14.54±2.3	24.63±4.53^##^
*t*_1/2β_ (h)	3.36±0.4	8.43±3.30*	3.79±1.24	6.58±4.5^#^
*Cl/f*(mL/min)	1.08±0.12	0.51±0.10**	0.7±0.10	0.54±0.27
*i.v. administration*				
*t*_1/2_ (h)	4.52±0.47	4.61±0.61	5.23±1.5	7.15±2.14
AUC (μg•mL^-1^•h)	28.1±1.00	27.14±2.58	29.55±1.23	30.83±3.12
CL/F (mL/min)	0.34±0.05	0.37±0.04	0.33±0.013	0.33±0.03
F（%)	33.3	74.6^*^	49.2	79.9^#^

* *p*< 0.05, ** *p* < 0.01 significant difference between parameters of enrofloxacin in the presence and absence of verapamil in 4 week old broilers. ^#^ *p*< 0.05, ^##^ *p* < 0.01 significant difference between parameters of enrofloxacin in the presence and absence of verapamil in 8 week old broilers.

### Effect of verapamil on pharmacokinetics of i.v. administered enrofloxacin

Because the plasma AUC is determined by both uptake and elimination, we also treated broilers with enrofloxacin given by i.v. injection. After i.v. administration, the maximum plasma levels of enrofloxacin were almost equal in the presence or absence of verapamil in both 4 and 8 week-old broilers (Figure 6). The effect of verapamil on the pharmacokinetic parameters is shown in Table 2. The combination of enrofloxacin/verapamil caused no significant changes in the pharmacokinetic behaviour of enrofloxacin. As shown in Table 2, the presence of verapamil did not significantly alter the t_1/2_, AUC and CL (*P*>0.05) of i.v. administered enrofloxacin, in broilers of the two chosen ages. Relative to their respective plasma AUC values after oral drug administration, the estimated bioavailability after oral administration (AUC_oral_/AUC_i.v._ ×100%) for 4 week-old broilers was 74.6% and 33.3% in the presence and absence of verapamil, respectively, and 79.9% and 49.2% in the presence and absence of verapamil, respectively, for 8 week-old broilers.

## Discussion

P-gp is a key molecule in determining not only the resistance of cancer cells against chemotherapeutic drugs but also the disposition of a variety of drugs in the intestine and other tissues [1]. Knowledge of ontogenic expression of P-gp involved in distribution and elimination of drugs is important to interpret the differences in therapeutic efficacy and safety between juvenile and adult animals. Age-dependent expression of P-gp in mammals has been extensively studied [10,12,31]; however, related information in chickens is not available. This prompted us to study the expression pattern of P-gp in the liver and different parts of the intestine of broilers at different ages (2~8 weeks old), corresponding to the stages in poultry husbandry. Here, for the first time, we show that the expression of P-gp mRNA peaked at 4 weeks, and then declined with age. Of greater importance, our results strongly suggest that the age-related expression of P-gp had a serious impact on the pharmacokinetics and bioavailability of oral enrofloxacin in broilers.

Examination of tissue-specific gene expression of P-gp together with its developmental difference is the first step to assess its physiological function in broilers. Our results of the organ and cellular distribution of P-gp in broilers were in agreement with previously reported findings in rodents and humans [32,33,34]. Limited studies have examined the ontogenic expression of P-gp in rats and chickens, but discrepant observations were reported [16,35,36]. Rosati et al. [36] found that *mdr1 a/b* level increased in liver and kidney in rats continuously until Day 60, then declined in all tissues with age, particularly during the period of 5-8 months old. In contrast, Kamath and Morris [37] found no difference in liver P-gp activity or protein level between young (22 days) and adult rats. Barnes [16] assessed P-gp expression in broilers from 0 to 21 days of age and found that it increased in the liver and kidney over the first few days of life with an apparent plateau at Day 2 and Day 4, respectively; however, the level in the duodenum did not significantly change with age. We found that P-gp mRNA expression was very low in the liver or small intestines in broilers from Day 1 to Day 7 (data not shown), and increased in a time-dependent manner at the early growing stage, peaking at 4 weeks of age in the liver, jejunum and ileum, before declining (Figure 1A). The ontogenic expression of P-gp in the duodenum reported in this study is in line with that published by Barnes [16], but the results for the liver are in sharp contrast to other reports. It is likely that this is due to different animals or feeds used. Further investigation is needed to address this issue.

The factors responsible for the age-dependent changes in P-gp expression are still unclear. However, Demeule et al. [38] and Iqbal et al. [39] have independently demonstrated that the expression of P-gp is up-regulated by steroids and other hormones whose activity may change during aging. Whether endogenous and/or exogenous (feed) hormones affect ontogenic P-gp expression in chickens needs further investigation. We speculate that the difference of P-gp expression peak between birds and mammals is probably related to the fact that birds consume a complex mixture of plant-based nutrients or non-nutritive material upon hatching, and thus birds might have earlier high levels of P-gp in response to the presence of substrates in the diet than mammals.

In addition, although enrofloxacin is available in an oral dosage form, the bioavailability of the oral form is lower than that of subcutaneous or intravenous forms in chicken, sheep and swine [40,41,42]; this has been ascribed to the metabolising enzymes within the intestines and the liver. Also, P-gp affects the absorption of enrofloxacin [43]. Considering that enrofloxacin is a substrate of P-gp [43], the modulation of P-gp expression level and activity may cause significant changes in the pharmacokinetic profiles of enrofloxacin. The C_max_, T_peak_, AUC and bioavailability of oral enrofloxacin in the broilers (4 and 8 week-old) in the current study were not in accordance with the values in other studies [40,44,45]. This might be due to different pharmaceutical formulations, or different ages or breeds of birds used in the studies. Li et al. [25] suggested that some excipients could enhance the absorption of a drug from the intestines by inhibiting P-gp. In order to preclude the effect of excipients on P-gp function, bulk drug of enrofloxacin was used in this study. The comparison of the enrofloxacin pharmacokinetics in 4 and 8 week-old broilers with different expression levels of P-gp strongly indicated the potential role of P-gp in enrofloxacin disposition. This is further supported by the finding that verapamil, an inhibitor of P-gp expression, was able to increase the absorption of enrofloxacin. Higher C_max_ and AUC of enrofloxacin after the co-administration of enrofloxacin/verapamil implied an increase in the absorption of enrofloxacin from the gastrointestinal tract. Although the expression of P-gp mRNA was also observed in the liver, the pharmacokinetics of enrofloxacin after i.v. administration alone or with the presence of verapamil were not significantly altered in both 4 and 8 week-old broilers. Therefore, the elevated bioavailability should result from the increased absorption of enrofloxacin by inhibiting P-gp in the intestine, rather than from decreased biliary excretion through inhibiting P-gp in the liver. Our results were somewhat similar to those reported by Sparreboom et al. [6], who found that the biliary excretion of paclitaxe was the same in P-gp knockout and wild-type mice. Seguin et al. [46] observed a poor oral bioavailability of enrofloxacin in neonatal kittens (2 to 8 weeks old), compared with that in adults; it is possible that the high level of P-gp in the intestine constituted a barrier that limited the absorption of enrofloxacin in kittens. Therefore, we should be aware that not all young animals should receive low doses under all circumstances. For some drugs (e.g. enrofloxacin), higher doses are actually needed to achieve therapeutic concentrations of the drugs in young animals.

In conclusion, age-related changes of P-gp expression in the intestine affect the pharmacokinetics of oral enrofloxacin in broilers. The higher expression of P-gp in the intestine of young broilers results in the low oral bioavailability of enrofloxacin. We expect that the results presented here for enrofloxacin are representative for other substrate drugs transported by P-gp. The findings also suggest that the co-administration of P-gp inhibitors may be an alternative strategy to improve the oral bioavailability and therapeutic efficacy of some other P-gp substrate drugs (e.g. ceftiofur licensed for use in animals) in young broilers. Further studies will focus on P-gp-associated different therapeutic efficiency of enrofloxacin in young and adult broilers challenged with *E. coli.*
